# The Curcumin Analog C-150, Influencing NF-κB, UPR and Akt/Notch Pathways Has Potent Anticancer Activity *In Vitro* and *In Vivo*

**DOI:** 10.1371/journal.pone.0149832

**Published:** 2016-03-04

**Authors:** László Hackler, Béla Ózsvári, Márió Gyuris, Péter Sipos, Gabriella Fábián, Eszter Molnár, Annamária Marton, Nóra Faragó, József Mihály, Lajos István Nagy, Tibor Szénási, Andrea Diron, Árpád Párducz, Iván Kanizsai, László G. Puskás

**Affiliations:** 1 AVIDIN Ltd., Szeged, Hungary; 2 Department of Pharmaceutical Technology, University of Szeged, Szeged, Hungary; 3 AVICOR Ltd., Szeged, Hungary; 4 Institute of Biochemistry, Biological Research Center of the Hungarian Academy of Sciences, Szeged, Hungary; 5 Institute of Genetics, Biological Research Center of the Hungarian Academy of Sciences, Szeged, Hungary; 6 Institute of Biophysics, Biological Research Center of the Hungarian Academy of Sciences, Szeged, Hungary; National Cancer Center, JAPAN

## Abstract

C-150 a Mannich-type curcumin derivative, exhibited pronounced cytotoxic effects against eight glioma cell lines at micromolar concentrations. Inhibition of cell proliferation by C-150 was mediated by affecting multiple targets as confirmed at transcription and protein level. C-150 effectively reduced the transcription activation of NFkB, inhibited PKC-alpha which are constitutively over-expressed in glioblastoma. The effects of C-150 on the Akt/ Notch signaling were also demonstrated in a *Drosophila* tumorigenesis model. C-150 reduced the number of tumors in *Drosophila* with similar efficacy to mitoxantrone. In an *in vivo* orthotopic glioma model, C-150 significantly increased the median survival of treated nude rats compared to control animals. The multi-target action of C-150, and its preliminary *in vivo* efficacy would render this curcumin analogue as a potent clinical candidate against glioblastoma.

## Introduction

Malignant glioma is the most common cancer of the central nervous system in adults with a median survival time of nine months following surgery, radiotherapy and chemotherapy. Despite advances in different therapies of glioblastoma, they are associated with significant side effects and only limited efficacy [1]. More effective therapeutic agents with fewer side effects are urgently needed.

Since cancer arises via multiple pathological or signaling pathways, natural compounds or their derivatives have the potential to be developed into optimum pharmaceuticals for cancer because of their multi-function and multi-target characteristics. Many pieces of evidence point out the relevance of herbal medicines for cancer therapy and prevention, including polyunsaturated fatty acids [2–4], corosolic acid [5] and dietary phytochemicals among others [6–8]. Recent attention has focused on curcumin, also known as diferuloyl methane, a polyphenolic, yellow pigment found in the rhizome of turmeric (*Curcuma longa*). It has been attributed antioxidant, anti-inflammatory, anti-angiogenic and anti-proliferative properties [9–10]. A number of preclinical and clinical studies suggest that curcumin may represent a novel strategy to treat cancer patients alone or in combination with already existing therapeutic regimens [9, 11]. Although recent advances in formulation show promise, the *in vivo* application of curcumin has been limited for its low potency and unsatisfactory pharmacokinetics [12, 13]. This necessitates the search for new formulation solutions and the synthesis of novel curcumin analogues with a similar safety profile, but improved pharmacological properties.

**Fig 1:**
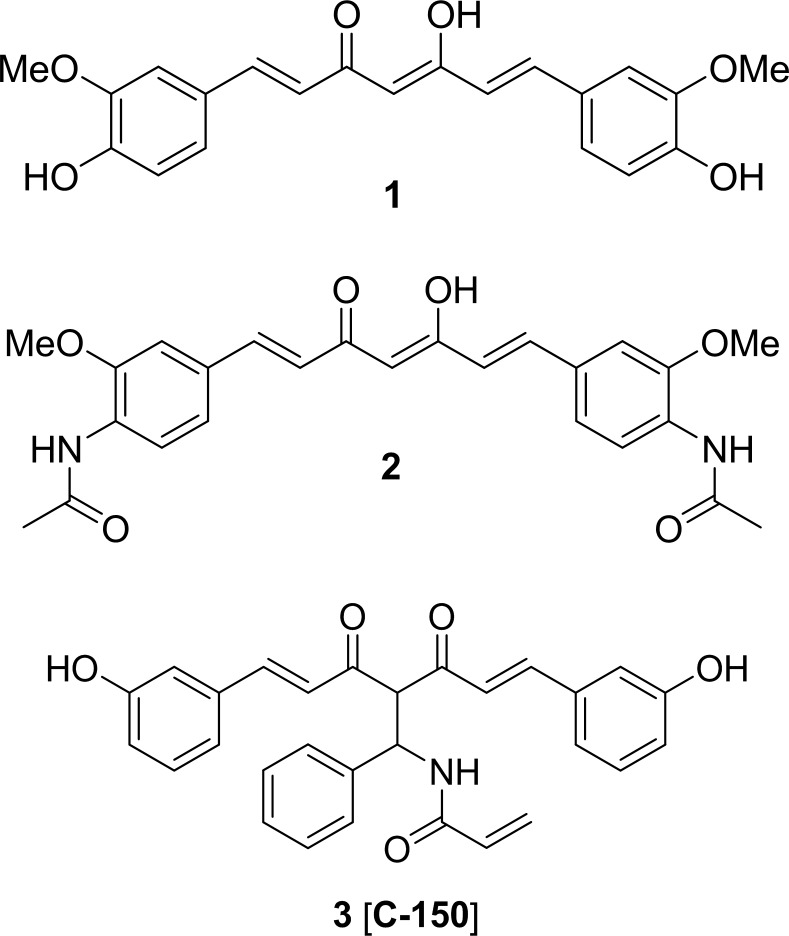
The chemical structure of curcumin and C-150. Curcumin **1**: (1*E*,4*Z*,6*E*)-5-hydroxy-1,7-bis(4-hydroxy-3-methoxyphenyl)hepta-1,4,6-trien-3-one, a related cytotoxic analogue that showed efficacy against glioma **2**, *N*,*N*'-(((1*E*,3*Z*,6*E*)-3-hydroxy-5-oxohepta-1,3,6-triene-1,7-diyl)bis(2-methoxy-4,1-phenylene))diacetamide and C-150 **3**, *N*-((*E*)-5-(3-hydroxyphenyl)-2-((*E*)-3-(3-hydroxyphenyl)acryloyl)-3-oxo-1-phenylpent-4-en-1-yl)acrylamide.

Different synthetic concepts have been therefore developed to expand the molecular diversity, from the side-chain and diketone transformations to alkyl and alkenyl functionalizations on C-4 in the central position of curcumin [14–16]. According to previous studies, modification at 4-position of curcumin enhanced its potency and selectivity and is playing a pivotal role in various biological activities including anti-inflammatory and anti-androgenic activities, and cytotoxicity [15]. Another series of curcumin analogues with different substituents at the 4-position of the phenyl group were synthesized and found active against human glioblastoma cell line [16]. Here, we describe a Mannich-type curcumin analogue C-150 (Fig 1), possessing meta-hydroxyphenyl side-chains and β-phenyl-β-acrylamido branched central motif, which was more potent than curcumin in suppressing proliferation of different glioma cell lines [17, 18].

The potential application of curcumin in neuro-oncology was suggested previously by authors who showed efficacy of this natural compound in cellular as well as *in vivo* models [19–25]. Curcumin has been shown to have multiple anticancer effects, including inhibition of proliferation, induction of apoptosis, and inhibition of angiogenesis [26, 27], but it also induces apoptosis-independent cell death [28]. Evidence indicates that the anticancer effects of curcumin and its structural derivatives are dependent on their capacity of modulating multiple molecular targets, including transcription factors, growth factors, kinases, inflammatory cytokines, adhesion molecules, apoptosis-related proteins [26], and signaling pathways such as NF-κB, AKT, MAPK, Wnt, Notch, p53 and Jak/Stat3 [24, 27, 29, 30]. Here, we demonstrated that C-150, a curcumin analogue also acts on multiple targets at the transcription and protein level at significantly lower concentrations than the original compound.

Tumor cells, including glioblastoma cells are more sensitive to endoplasmic reticulum (ER) stress inducing agents than normal cells as their ER-stress response is continuously engaged, due to their chronic stress situation, leading to activation of pro-apoptotic signals and finally tumor cell death. Preclinical development of novel anti-glioma drugs targeting the ER-stress response has been recently reviewed [19]. In this paper fast activation of ER-stress by curcumin and its derivative was confirmed by inducing GRP78 and GADD153 protein expression.

Curcumin has been shown to increase the concentration of reactive oxygen species which causes ER-stress, and caspase 3-dependent and -independent apoptosis through the release of cytochrome C and apoptosis inducing factor from the mitochondria, and inhibition of AKT [24, 31]. AKT is a downstream serine/threonine kinase in the RTK/PTEN/PI3K pathway. The activated AKT and phospho-AKT levels are elevated in the majority of glioblastoma cell lines and tumor samples, therefore inhibitors of the Akt pathway represent a potential treatment option against glioblastoma [32]. It was also suggested that curcumin inhibition of AKT/mTOR/S6K signaling pathway may be related to its induction of autophagy in malignant glioma cells [33].

The effects of C-150 on the Akt/Notch signaling were also demonstrated in a *Drosophila* cancer model, where malignant transformation occurs in the eye based on a double Akt/Notch „gain-of-expression” mutant strain [34]. This *in vivo* model can be used for testing curcumin analogs for their potency on modulating the Akt/Notch signaling, and could provide their first *in vivo* proof of concept results for potential anticancer agents. Since, this model cannot be translated directly to human therapy, mainly because of the differences in ADME parameters we also investigated the effects of C-150 in an *in vivo* glioma model.

## Results and Discussion

### Cytotoxic effects of C-150 in glioblastoma cells

**Fig 2:**
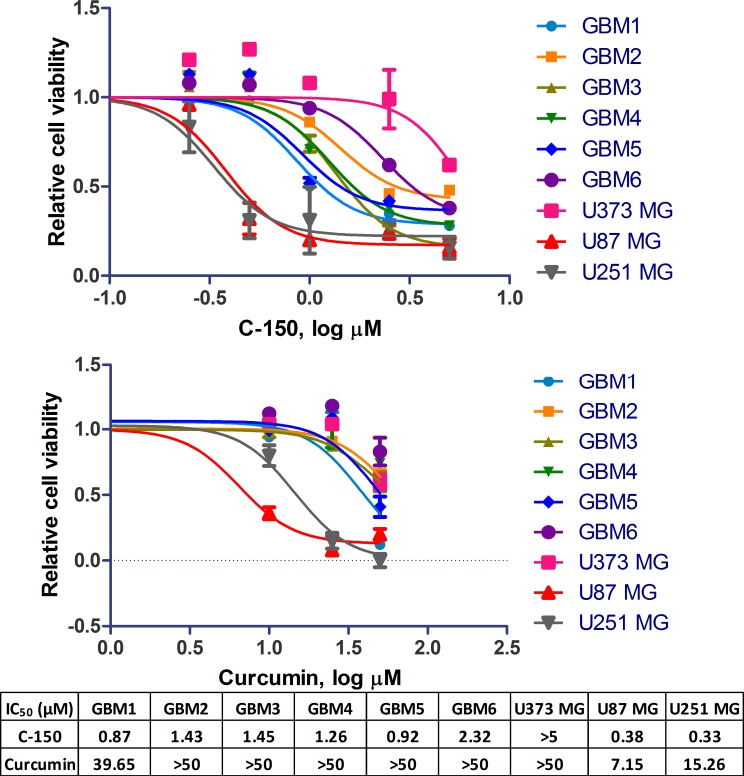
Cytotoxic effects of curcumin and C-150 on nine different human glioma cell lines determined by MTS assay. IC_50_ values (μM) for C-150 and curcumin are indicated in the table.

The cytotoxicity of C-150 curcumin analogue was assessed in a panel of human glioblastoma cell lines (namely: GBM1-6, U87 MG, U251 MG and U373 MG) using the MTS assay. IC_50_ values for 48 h exposure were summarized in Fig 2. Although the effect of curcumin was reported previously on a few glioma cells, it was included here to evaluate its cytotoxicity in other glioma cells and to provide a comparison with the new analogue.

C-150 exhibited pronounced cytotoxic effects against all glioma cell lines at micromolar concentrations. Between the most sensitive cell line U251 MG (IC_50_: 0.33 μM) and the most resistant cell line U373 MG (IC_50_: >5 μM) there was at least a 15-fold difference in case of C-150.

**Fig 3:**
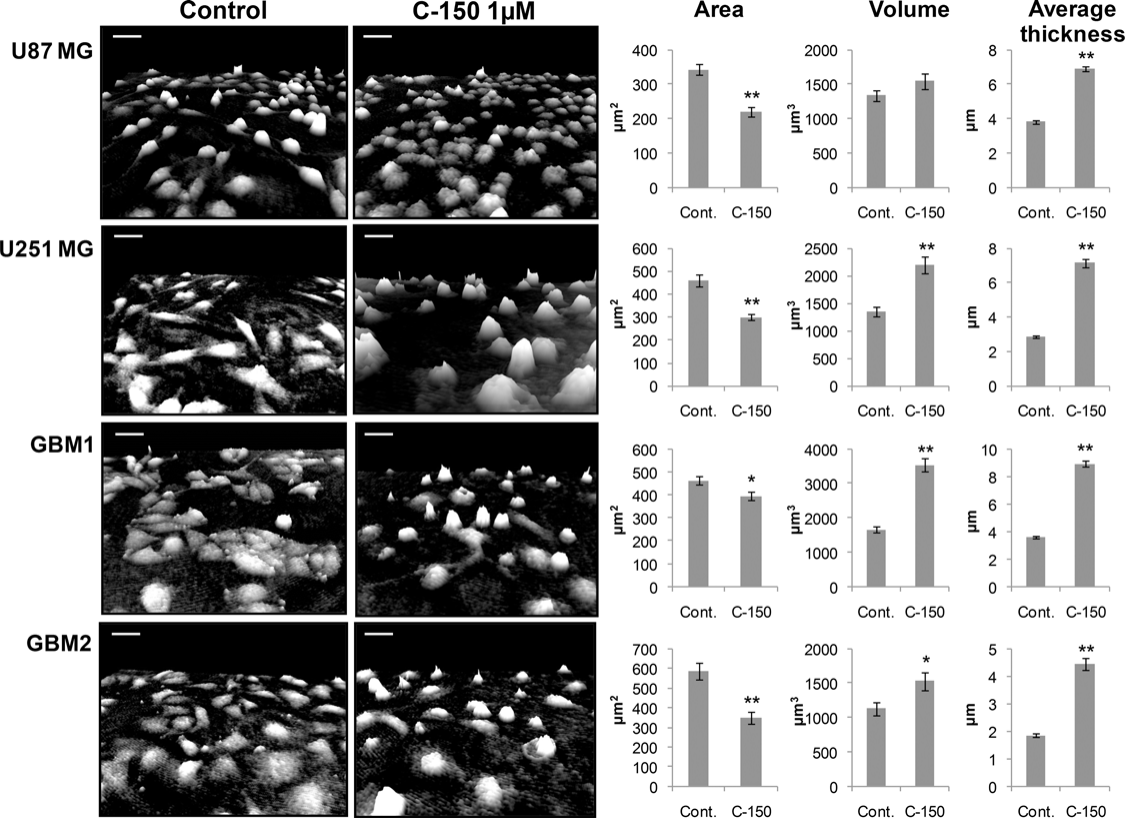
Holographic microscopic analysis of C-150 induced cell death. Tumor cells (U87 MG, U-251 MG, GBM1, GBM2) were treated with 1 μM C-150. Cell volume, area and average thickness data are presented for each cell line 24 h after treatment. Error bars represent the standard error of means (SEM). Statistical significance:* p<0.05 and ** p<0.01 (unpaired, two tailed Student’s t-test).

To visualize the cytotoxic effects of C-150 holographic microscopy analysis (HoloMonitor 3M, Phiab, Sweden) was also performed [35, 36]. This technology is label-free and non-invasive, it detects only the effects of the tested compound [37]. These experiments record morphological parameters of treated cells such as area, thickness and volume. We incubated four different glioma cell lines (U87 MG, U-251 MG, GBM1, GBM2) with 1.0 μM C-150 and holographic images were taken before and 24 h after treatment. C-150 induced cell death after 24 h treatment is illustrated with holographic images in Fig 3. Decrease in cellular area and the increase of average thickness could be registered after treatment, in case of all four treated cell lines as a hallmark of cell death (cell rounding and detachment from the surface, Fig 3). It is the first time where the effects of drug induced cell death on cellular morphology of these glioma cells were detected by using 3D holographic images.

### Multiple targets of C-150

#### Biochemical and *in vitro* assays

Curcuminoids have been reported to interact with multiple molecular targets, such as NF-kB, STATs, AP-1, PPAR-g, etc. [26, 27, 29, 30]. Our aim was to compare the effects of curcumin and C-150 on different cellular targets.

**Fig 4:**
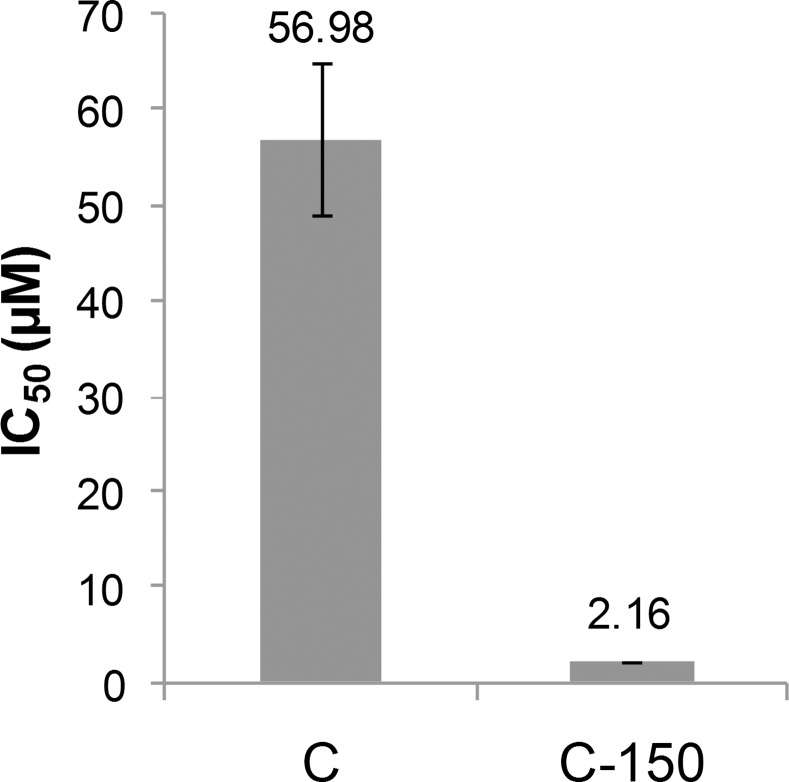
Inhibitory effect of curcumin or C-150 on NF-κB activation. IC_50_ values obtained in an *in vitro* NF-κB activation inhibition assay (C: curcumin, C-150: novel curcumin analog). Error bars represent standard deviation from repeated experiments.

Curcumin was found to be a potent inhibitor of the activation of nuclear factor-kB (NF-κB) and activated protein-1 (AP-1) in glioblastoma cells [21]. NF-κB proteins influence the expression of genes that are involved in a large number of physiological processes including immune response, cell survival, differentiation, and proliferation [38]. One of the predominant targets of curcumin is the NF-κB cell signaling pathway [39]. Moreover, correlation between NF-κB inhibitory potential of different curcumin analogs and their cytotoxicity has been recently reported [40]. In our study using a TNFα-induced luciferase gene expression model, the effects of C-150 and curcumin was studied (see Fig 4). C-150 exhibited a very potent inhibition: it inhibited NF-κB activation at micromolar concentration (IC_50_ = 2.16±0.02 μM). This value is 26-times lower than that of curcumin (IC_50_ = 56.98±7.79 μM). Similar differences in cytotoxic potential could be recorded between curcumin and its derivative C-150 against glioma cells (see Fig 2).

There is increasing evidence that ER stress plays a crucial role in the regulation of apoptosis. Recent studies suggested that curcumin-induced apoptosis may be mediated by oxidative and ER stress signaling in several cancer cell types [41, 42]. The unfolded protein response (UPR) consists of a set of adaptive pathways that are triggered by different perturbations in normal function of the ER that lead to the production of misfolded proteins. The UPR alleviates ER stress by up-regulation of chaperones and folding enzymes, degradation of misfolded proteins, and arresting general translation. During this response, several pro-survival and pro-apoptotic signals are activated and depending on the extent of the ER stress, cells survive or when ER functions are severely impaired, they undergo apoptosis [43]. The UPR involves the activation of several proteins, including the glucose-regulated protein 78 (GRP78/BiP), which represents the pro-survival arm of the UPR. On the other hand, the CCAAT/enhancer binding protein (CHOP/GADD153) transcription factor is one of the critical executioners of the pro-apoptotic arm of the UPR [44]. When cells are experiencing ER stress GADD153 is induced and hence initiating the cell death process.

**Fig 5:**
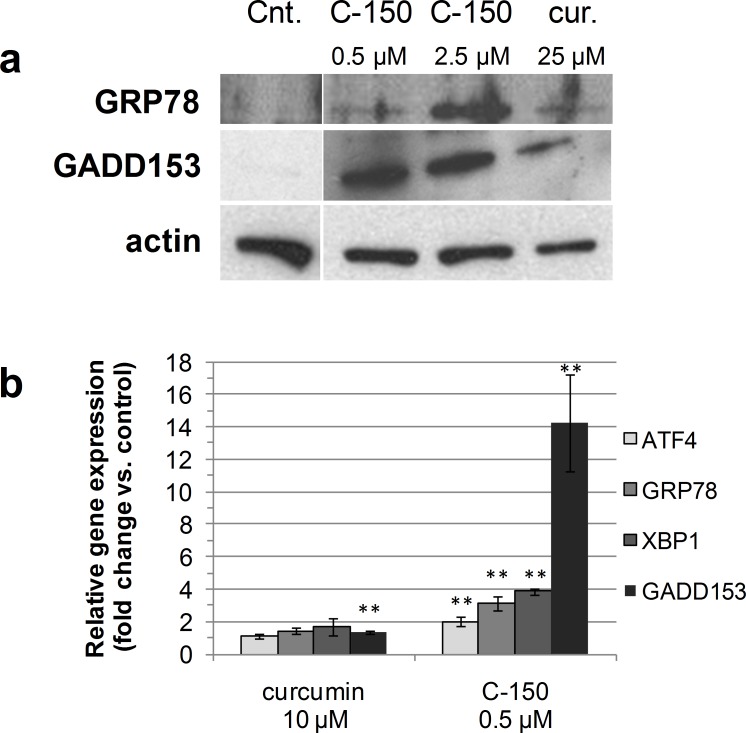
Effects of C-150 and curcumin on protein and gene expression. Effects of C-150 and curcumin on GRP78 and GADD153 protein expression (**a**). U87 MG glioma cells were treated with compounds at the indicated concentrations for 6 h. Cells were collected and the total lysates isolated and examined by Western blot analysis using an anti-GRP78 or anti-GADD153 specific antibody. Actin is shown as a control for equal loading. (**b**) Induction of mRNA levels of genes involved in UPR and ER-stress (ATF4, XBP-1, GRP78 and GADD153) as verified by using QRT-PCR method in U87 MG cells. Fold changes are shown for curcumin (10 μM) and C-150 (0.5 μM) treated cells 6 h post-treatment, relative to untreated controls. Statistical significance: * p<0.05 and ** p<0.01

As shown in Fig 5A, protein expression of GRP78 was increased 6 h after C-150 treatment, while a significant increase could be observed in the curcumin-treated group at 25 μM concentration, which is approximately 50-times the dose of C-150. Similar values were reported by Pae *et al*. They found that curcumin-induced apoptosis of HL-60 leukemia cells was associated with modulation of ER stress-related proteins after treatment with curcumin at 20 μM [42].

Western blotting analysis further showed that GADD153 protein expression was markedly increased 6 h after C-150 treatment. It can be seen in Fig 5A, that C-150 induced GADD153 protein expression in a dose-dependent manner. Similarly, GADD153 expression was also detected in curcumin-treated cells at 25 μM concentration. Scott and his co-workers also indicated that curcumin induced GADD153 upregulation in colon cancer cells at 20 μM concentration [45].

The effect of curcumin and C-150 on GRP78, the protective component of the UPR, as well as on GADD153, the pro-apoptotic arm of the UPR, were examined by QRT-PCR to determine whether these compounds would be implicated in ER stress or UPR levels. As shown in Fig 5B, GRP78 was up-regulated by an average of 3-fold, while GADD153 expression was induced by 14-fold in C-150 treated U87 MG cells, whereas no induction of these genes was detected in case of curcumin at 10 μM. The activity of the pro-survival and pro-apoptotic component suggest that in these conditions the cell survival signals mediated by GRP78 were overwhelmed by the activity of the pro-apoptotic GADD153 activity sending the cells toward an apoptotic fate. Similar observations were described in a rat retinal detachment model by Liu and coworkers [46].

Several factors are known to be responsible for ER stress mediated transcriptional activation of GRP78, including XBP-1 and activated ATF-4. ATF-4 is a cAMP response element-binding transcription factor (CREB) and promotes cell survival by activating certain gene transcription including those involved in the UPR and ER stress response [47].

When we investigated whether XBP-1 and ATF-4 transcription was affected by C-150, we found that 0.5 μM C-150 treatment up-regulated ATF4 2-fold and XBP-1 by almost 4-fold at the mRNA level. We then tested whether this observation was applicable to 10 μM curcumin, as well, but no induction could be registered for either of these genes at this concentration.

In summary, the ER could be a direct target site of C-150 or C-150 may indirectly elicit ER stress acting through other cellular pathways. Our results demonstrated the capacity of C-150 to activate key genes and their protein products in initiation and execution phases of ER stress and subsequently apoptosis at low- and sub-micromolar concentrations.

**Fig 6:**
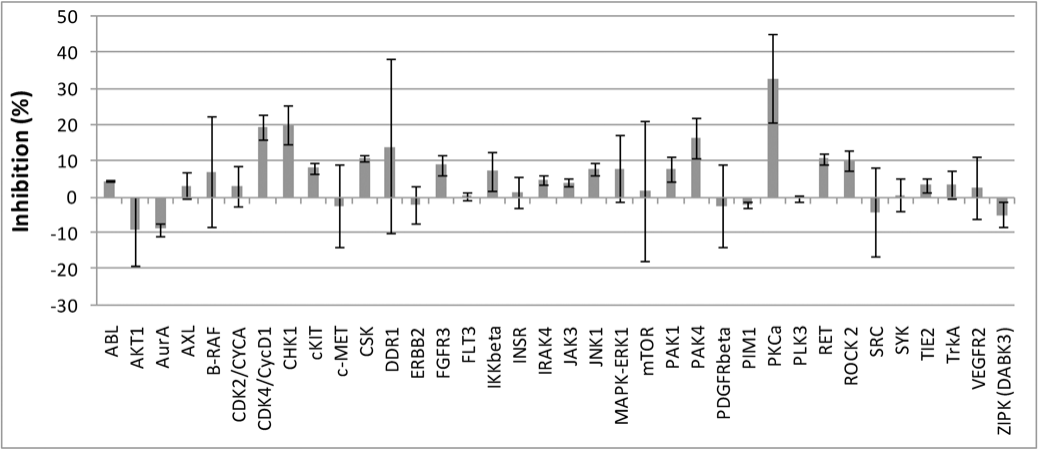
Kinase profiling of C-150. The effects of C-150 (1μM) on kinase inhibition was examined on a kinase panel of numerous different kinases. Note the significant inhibition of PKC-alpha.

The effects of C-150 on kinase inhibition was also studied on a kinase panel consisting of 28 different kinases (RET, ROCK 2, SRC, SYK, TIE2, mTOR, PAK1, PAK4, PDGFRbeta, PIM1, IKKbeta, IRAK4, InsR, JAK3, JNK1, c-MET, CSK, DDR1, ERBB2, FGFR3, B-RAF, CDK2/CYCA, CDK4/CycD1, CHK1, cKIT, ABL, AKT1, AURA, AXL, FLT3, VEGFR2, TrkA, PKC-alpha) (Fig 6). Preliminary screening of C-150 was performed at 1 μM concentration to assess possible specific kinase inhibition. Under this condition C-150 exhibited significant inhibition (32.8%) only with PKC-alpha. This result of course, does not mean that C-150 does not interact with other kinases in the panel at higher concentrations, or other kinases which are not present in the list. Among many intracellular signaling proteins, protein kinase C (PKC) isoenzymes have been identified as possible drug targets against tumor cells. PKC-alpha expression in nervous system tumors, including glioblastoma has been reviewed recently [48]. In pre-clinical experiments aprinocarsen, an antisense oligonucleotide targeting PKC-alpha had demonstrated anti-tumor activity, in particular in animal models of glioblastoma. The inhibition of PKC-alpha by C-150 could act synergistically with its other targets. This multi-target action would render this curcumin analogue a potent clinical candidate against glioblastoma as tumor cells cannot develop resistance easily.

#### *In vivo* target validation

**Fig 7:**
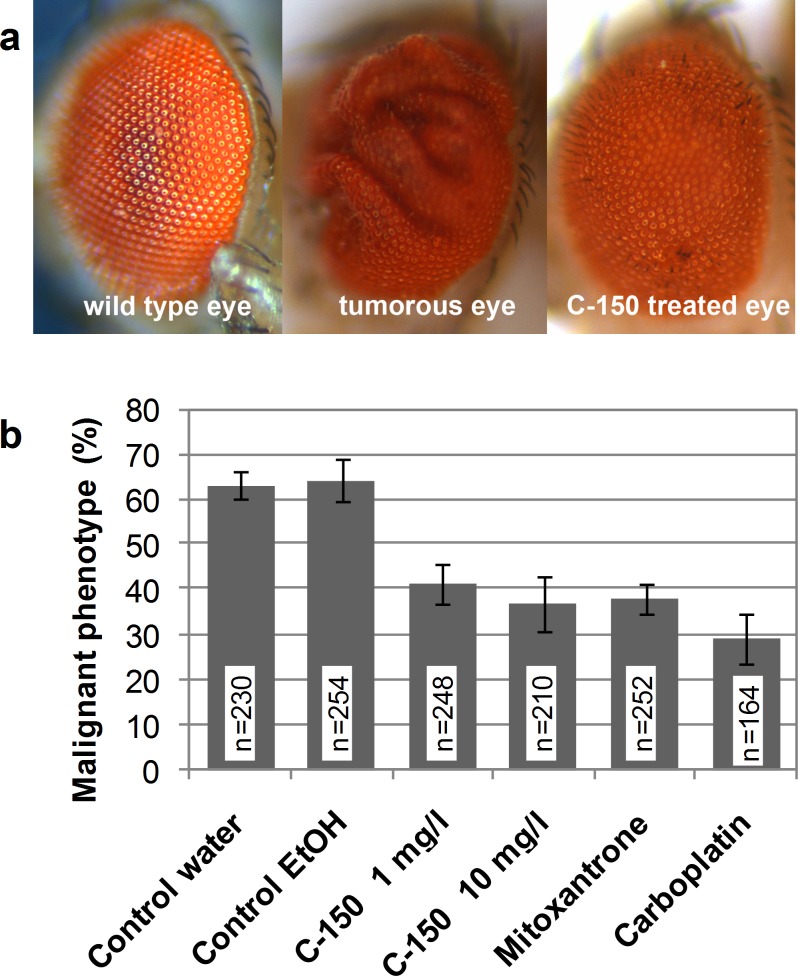
*In vivo* effect of C-150 on a *Drosophila* double Akt/Notch „gain-of-expression” mutant strain. (**a**) Malignant transformation occurs in the eye. (**b**) Tumor incidence is presented for each control and treated group (sample size is indicated for each group overlaid on the columns). Error bars represent standard deviation from 3 independent measurements. All treatments had a p value below 0.01 when compared to their respective controls (Student’s t-test).

The activated AKT pathway supports glioma cells to evade apoptosis and enhance their invasion potency, by amplification of growth signals, thereby making inhibition of AKT an attractive target for chemotherapy against glioblastoma [32, 33]. Several studies reported that curcumin inhibits cancer cell proliferation by modulating Akt/mTOR signaling [33, 49]. A recent study by Li et al. suggested that activity of curcumin is at least partly mediated through inactivation of the Notch signaling pathway [50]. Notch signaling and the PI3K-AKT pathway has been shown to synergize *in vivo* in a Drosophila melanogaster model of Notch-induced tumorigenesis [34]. When the Notch ligand Delta was overexpressed, and the „gain-of-expression” mutation of the Akt1 gene was also present in Drosophila tumors developed in the eye. We used this model to confirm the effects of curcumin analogue, C-150 on modulating the Notch/Akt signaling pathway *in vivo*. Under our experimental conditions about 60% of the animals kept on media having either water or ethanol developed tumors (58.7%, n = 104; 61.7%, n = 162, respectively) (Fig 7). When mitoxantrone or carboplatin (1 mg/l) was applied a significant reduction of tumor-bearing animals could be recorded (37.6%, n = 178, and 28.3%, n = 92, respectively). C-150 was dissolved in ethanol and mixed into the medium at two concentrations (1 mg/l and 10 mg/l). Mutant Drosophila flies were grown on supplemented media and malignant phenotype was recorded. As seen in Fig 7 C-150 resulted in concentration-dependent reduction of tumor occurrence. At lower concentration C-150 decreased tumor incidence by almost 30% (malignant phenotype was recorded in 42.9% of the animals; n = 182), while 10 mg/l C-150 resulted in 42% decrease when compared to control groups (35.6% tumor incidence, n = 132).

### *In vivo* effects of C-150 in orthotopic glioma xenograft

The *in vivo* effectiveness of C-150 was tested against intracerebrally implanted human glioblastoma cells (U87 MG) in the nude rat xenograft model. To circumvent bioavailability problems, we used a liposome delivery system for the encapsulation of C-150 and intravenous administration.

**Fig 8:**
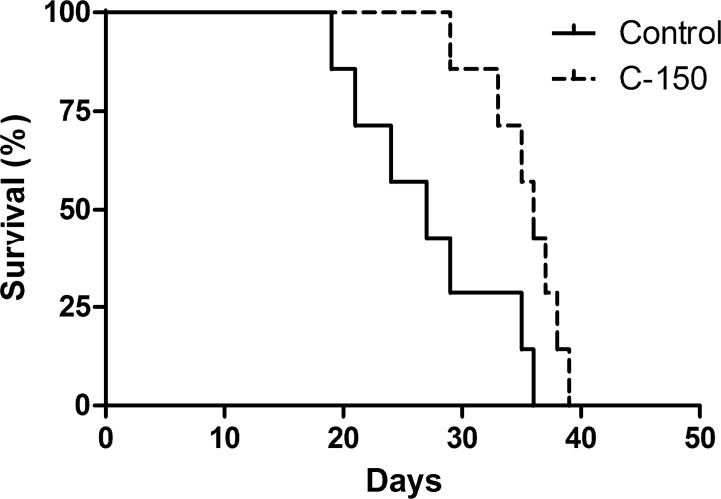
*In vivo* effects of C-150 in orthotopic glioma xenograft. Kaplan-Meier survival curves of rats intracerebrally grafted with U87-MG cells and treated with C-150. Time is expressed in days from inoculation. Animals treated with C-150 displayed a significantly longer median survival time compared to control animals (27 vs. 36 days, n = 7 in both groups, p = 0.0181 Log-rank (Mantel-Cox) Test).

Animals treated with C-150 displayed a significantly longer median survival time (27 days) compared to control animals (36 days, p = 0.0181) (Fig 8).

## Conclusions

Malignant gliomas are associated with a poor prognosis due to inherent drug and radiation resistance, therefore more effective therapeutic agents with less side effects are urgently needed. The present study demonstrated a potent, cell death-inducing effect of C-150, an analogue of the natural compound, curcumin, in human glioma cells.

Cytotoxic effects of C-150 were mediated by affecting multiple targets as confirmed at transcription and protein level. C-150 effectively reduced the transcription activation of NF-κB in an *in vitro* model, a result that may be beneficial in glioblastoma where NF-κB is constitutively over-expressed. C-150 also decreased the expression of genes implicated in cytoprotection and chemo-resistance [51, 52]. Curcumin has been previously shown to activate UPR and induce ER-stress and subsequent apoptosis of tumor cells. Here we demonstrated that C-150 was at least 30-times more potent than curcumin to induce the expression of key genes and their protein products in initiation and execution phases of ER stress at low- and sub-micromolar concentrations. In a kinase panel consisting of 28 different kinases C-150 inhibited PKC-alpha, a kinase that has been implicated in tumors of the CNS, including glioblastoma. Among the targets of curcumin, the Akt/mTOR and Notch pathways have preferential roles in cancer cell proliferation. C-150 was able to decrease *Drosophila* tumorigenesis in their eye in a double Akt/Notch „gain-of-expression” mutant strain verifying that this pathway is modulated by C-150, although the affected targets seem to be other than mTOR and AKT1, since the biochemical assay testing kinase inhibition showed no effect in case of these two kinases. Finally, C-150 significantly increased the survival of treated rats in a glioma animal model compared to vehicle control.

Increased potency compared to curcumin, the demonstrated multi-target action and the demonstrated *in vivo* activity of C-150 would render this analogue a potent clinical candidate against glioblastoma.

## Materials and Methods

### Cell culture studies

Glioblastoma cells (U373 MG, U87 MG, GBM1-GBM6 cell lines were a kind gift from Balazs Hegedus, Semmelweis University, Hungary, [53] while the U251 MG cell line was a gift from Szabolcs Bellyei, University of Pecs, originally obtained from American Type Culture Collection, Manassas, VA) were grown at 37°C under 5% of CO_2_ and 100% humidity in DMEM and RPMI medium supplemented with 10% fetal calf serum (FCS) (Sigma-Aldrich), and penicillin-streptomycin antibiotics. For cytotoxicity assays, 10,000 cells were seeded into each well of 96-well cell culture plates in culture medium containing 10% FCS. Effects of curcumin and C-150 were recorded 48 h after treatment. MTS (3-(4,5-dimethylthiazol-2-yl)-5-(3-carboxymethoxy-phenyl)-2-(4-sulfophenyl)-2H-tetrazo-lium) reagent was applied to drug treated and control (0.2% DMSO) cells (CellTiter 96® AQueous Assay, Promega, Madison, WI) according to the manufacturer’s protocol.

### Holographic cell analysis

Digital holographic images were taken by the HoloMonitor M3 instrument (Phase Holographic Imaging AB, Phiab, Sweden). Four different glioma cell lines (U87 MG, U-251 MG, GBM1, GBM2) were treated with 1.0 μM C-150 in T25 flasks and incubated for 24 hours. Holographic images were captured of the same culture area before and after treatment. Cell morphological changes were analyzed by Holostudio 2.4 software (Phase Holographic Imaging AB, Phiab, Sweden). Each point in the scatter plot represents data of a single cell.

### Western blot analysis

U87 MG cells were lysed in RIPA buffer (25 mM Tris-HCl pH 7.6, 150 mM NaCl, 1% NP-40, 1% sodium deoxy-cholate, 0.1% SDS) containing 1% (v/v) mammalian protease inhibitor cocktail from Sigma and separated on 10% SDS-PAGE, following by transferring to a nitrocellulose or PVDF (Immobilon-P Transfer Membranemembrane, Millipore). The membrane was then blocked in blocking buffer (20 mM Tris-HCl, 150 mM NaCl, 0.1% Tween-20, 10% milk) for overnight (16 hours) at room temperature. After blocking, corresponding primary antibody was incubated for one hour, followed by a 45 minute incubation with the corresponding HRP-conjugated secondary antibody at room temperature. Extensive washes with a blocking buffer were performed between each step. The protein immuno-complex was visualized by SuperSignal West PICO Chemiluminescent Substrate (Thermo Scientific). Dilutions for the primary antibodies were as follows: anti-GADD 153 mouse monoclonal antibody (sc-7351, Santa Cruz) at 1:200, anti-GRP78 rabbit polyclonal antibody (sc-13968, Santa Cruz) at 1:1000, anti-p-Akt1/2/3 (Ser 473) rabbit polyclonal antibody (sc-7985, Santa Cruz) at 1:200, anti-β-actin mouse monoclonal antibody (sc-47778, Santa Cruz) at 1:200.

### Gene-expression analysis by QRT-PCR

Total RNA was purified from 400,000 of curcumin, C-150 treated and control (0.2% DMSO) cells (6 h after treatment) with AccuZol^TM^ RNA purification kit (Bioneer, Daeleon, Korea) according to the manufacturers’ protocol. Three independent treatments were carried out with each condition. Total RNA (2 μg) was converted into cDNA with the High-Capacity cDNA Archive Kit (Applied Biosystems, Foster City, CA) in a total volume of 20 μl, and without purification the mixture was diluted five times and 2 μl was applied to QRT-PCR analysis. QRT-PCR was performed on The LightCycler® Nano Instrument (Roche). Expression of S18 rRNA and GRP78 were measured with corresponding TaqMan Gene Expression Assays (Life Technologies, Cat. # 4331182 and Cat. # 4331182), while GADD153, ATF4 and XBP1 expression were measured with the SybrGreen protocol using the following primers: GADD153_F: 5’-cagagctggaacctgaggag-3’; GADD153_R: 5’-tggatcagtctggaaaagca-3’; ATF4_F: 5’-tctccagcgacaaggctaa-3’; ATF4_R: 5’-caatctgtcccggagaagg-3’; XBP1_F: 5’-ggagttaagacagcgcttgg-3’; ATF4_F: 5’-tctccagcgacaaggctaa-3’; ATF4_R: 5’- caatctgtcccggagaagg-3’; XBP1_R: 5’-cactggcctcacttcattcc-3’ [54]. Relative expression ratios were normalized to S18 rRNA. For significance analysis Student’s t-test was applied in Excel.

### NF-κB Activation Assay

The B16 cell line was maintained in RPMI medium (Lonza) supplemented with 10% FCS (Lonza). The NF-κB reporter cell lines were created by transfection with the pNF-κB-Luc/neo reporter construct with the Lipofectamine 2000 reagent (Invitrogen) [55]. Stable cell lines were selected by G418 (Sigma) treatment.

B16/NF-κB-Luc cells, grown on luminoplates (Corning-Costar) were used. Cells (5×10^4^ cells/well) were treated with C-150 or curcumin (or vehicle control) after 30 min of NF-κB induction by TNFα (10 ng/ml). After 6 hours of incubation with inducers, the medium was removed, the cells were washed and lysed for 10 min at room temperature in Cell Culture Lysis Reagent (20 μl/well; Promega). Substrate was added (20 μl/well; Promega), and luciferase activity was measured in a Luminoskan Ascent (Thermo Electron Corporation) scanning luminometer. Cell viability was routinely determined using trypan blue exclusion test during the assays to make sure that assays were always carried out on viable cells.

### Kinase profiling of C-150

Selectivity panel measurements were performed by Proteros biostructures GmbH (Germany) on selected kinases. Assays were based on either IMAP-FP (Molecular Devices) or Reporter Displacement Assay (RDA) (Proteros) [56]. In cases of b-Raf, DDR1 and mTOR RDA were used, in other cases IMAP-FP were applied. Protocol for IMAP-FP (substrate finder) assays: reaction volumes were 8 μl/well in a 384-well black plate. Final assay concentrations: kinase concentration yielding 50% substrate turnover. ATP: adjusted to Km_app_. Substrate: 400 nM, type of substrate was adjusted to different kinases. Protocol for RDA assays (a high-throughput binding assay for affinity, kinetic and thermodynamic profiling): reaction volumes were 15 μl/well in a 384-well black plate. Final assay concentrations of the kinases: b-Raf 9.0 nM, DDR1 15.0 nM, mTOR 8.0 nM. Reporter Probe: adjusted to reporter probe Kd. C-150 was screened at 1 μM concentration.

### Fly stocks and drug treatment

To generate *ey-Gal4<UAS-Dl/+; GS1D233C(Akt)/+* flies, frequently exhibiting eye tumors, we crossed *w*^*1118*^*; ey-Gal4<UAS-Dl/CyO*, *twi-GFP* females to *w*^*1118*^*; GS1D233C(Akt)/TM3*, *twi-GFP* males. From the progeny of this cross we collected the freshly hatched “non-green” larvae that were distributed into test vials containing 5 ml of standard fly food supplemented with C-150 dissolved in ethanol or the corresponding solvent as control. Each chemical was tested in at least three independent vials, eye phenotypes were analyzed in few days old adult flies. For significance analysis Student’s t-test was applied in Excel.

### Liposome encapsulation of C-150

Liquid-phase C-150 containing liposomes were prepared by dissolving lipid powder CHOL/PC/DSPE-mPEG (62/28/0.6 mol%) and C-150 (9.2 mol%) in EtOH. After the solvent had been removed, the lipid film was hydrated and redispersed in PBS solution to a final C-150 concentration of 1 mg/ml. The dispersion was subjected to size extrusion (0.45 μm pore size) and finally filtered through sterile filters (0.22 μm pore size). The droplet size, SSA and PDI of the liposomes were measured by laser diffractometry (n = 5) by the wet method. The d(0.1), d(0.5) as median and d(0.9) droplet sizes, SSA and PDI were 75 ± 19 nm, 114 ± 20 nm, 178 ± 24 nm, 54.5 m2/g and 0.901 ± 0.20, respectively.

### *In vivo* orthotopic glioma xenograft

Female nude rats (RNU rats, Charles River, Hungary) were housed in sterile cages. They were fed autoclaved food and sterile water *ad libitum*. For inoculation U87 MG cells were trypsinized, washed and resuspended in sterile PBS. 10^6^ Cells were injected intracerebrally. All animals were treated moribund and were euthanized at the observation of the first sign of torment. All operative procedures and animal care conformed strictly to the Hungarian Council on Animal Care guidelines.

Animals were randomized and two groups, control (n = 7) and treated (n = 7) were created. The administration of C-150-containing liposomes into the tail veins started on day 4 and was continued 3 times a week for 4 weeks at one-sixth of the tolerated dose: 3 mg/kg. For significance analysis Log-rank (Mantel-Cox) test was applied in GraphPad Prism.

## Supporting Information

S1 Table QRT-PCR results. Table showing all of the QRT-PCR experiments performed with C-150 at various concentrations. The second table shows statistical significances of corresponding gene expression changes (Student’s t test).
(PDF)
