# Supplementary material for: The Curcumin Analog C-150, Influencing NF-κB, UPR and Akt/Notch Pathways Has Potent Anticancer Activity In Vitro and In Vivo
Source: PLoS One. 2016 Mar 4;11(3):e0149832. doi: 10.1371/journal.pone.0149832 (PMC4778904; doi:10.1371/journal.pone.0149832)
Supplement: S1 Table — Table showing all of the QRT-PCR experiments performed with C-150 at various concentrations. The second table shows statistical significances of corresponding gene expression changes (Student’s t test). (PDF) [file pone.0149832.s001.pdf]

Supplementary Table 1.

	ATF4		GRP78		XBP1		GADD153	
	Fold change	SD	Fold change	SD	Fold change	SD	Fold change	SD
**Control**	1.00	0.10	1.01	0.17	1.00	0.12	1.00	0.07
**C, 10 μM**	1.10	0.16	1.42	0.21	1.69	0.56	1.36	0.05
**C-150, 0.5 μM**	2.03	0.30	3.12	0.44	3.88	0.19	14.24	2.95
**C-150, 1 μM**	2.18	0.28	4.83	0.13	3.74	0.51	10.24	2.16

p values (n=3)	ATF4	GRP78	XBP1	GADD153
**C 10 μM vs Control**	0.43275	0.06132	0.10528	0.00290
**C-150, 0.5 μM vs Control**	0.00256	0.00093	0.00005	0.00003
**C-150, 1 μM vs Control**	0.00125	0.00010	0.00024	0.00005
